# Effect of Soursop (*Annona muricata*) Puree and Gum Arabic From *Acacia senegal* var. *kerensis* on the Physicochemical and Nutritional Properties of Non‐Dairy Coconut (*Cocos nucifera*) Milk–Based Ice Cream

**DOI:** 10.1002/fsn3.70848

**Published:** 2025-08-27

**Authors:** Sarah Nura Mulwa, Symon Maina Mahungu, Benard Kimatu Muinde

**Affiliations:** ^1^ Department of Dairy, Food Science and Technology Egerton University Njoro Kenya

**Keywords:** coconut milk–based, gum arabic, ice cream, nutritional, physicochemical, soursop puree

## Abstract

Plant‐based milks are increasingly popular in producing ice cream and frozen desserts as dairy alternatives. Their distinct nutritional and physicochemical characteristics affect the final product. This study aimed to incorporate soursop fruit puree and gum arabic from *Acacia senegal* var. *kerensis* into a nondairy coconut milk–based ice cream and examine their effects on the product's properties. Physicochemical analyses, including overrun, melting rate, pH, and total soluble solids (TSS), were performed on the ice cream samples. The nutritional analysis followed specified AOAC methods for moisture, proteins, fats, ash, crude fiber, vitamin C, total phenolics, and minerals (calcium, iron, zinc, sodium, magnesium, and potassium). Data analysis utilized SAS program version 9.1.3 for ANOVA and least squares means, with soursop concentrations at 0%, 10%, 20%, and 30% and gum arabic levels from 0% to 1.5%. A control variant with gelatine and carboxymethylcellulose (CMC) was also prepared. Duncan's multiple range test (DMRT) was applied postsignificance at *p* < 0.05. Results showed that ice cream with 20% and 30% soursop achieved the highest overrun (20.01% and 26.99%) and the lowest pH (4.61 and 4.30), alongside reduced melting rates (0.10 and 0.09 g/min, respectively). Increased overrun correlated with higher gum arabic levels. TSS values peaked at 1% and 1.5% gum arabic (33.83° and 33.81°Bx, respectively). The addition of soursop and its interaction with gum arabic significantly impacted protein, fat, ash, fiber, moisture, vitamin C, and minerals (*p* < 0.05). This study demonstrated that incorporating 30% soursop and 1% gum arabic improves the physicochemical and nutritional properties of coconut milk–based ice cream, presenting a consumer‐preferred alternative.

## Graphical abstract

This study investigated the incorporation of soursop puree and gum arabic into nondairy coconut milk–based ice cream and evaluated their effects on physicochemical and nutritional properties. Ice cream containing 30% soursop and 1% gum arabic demonstrated improved overrun, reduced melting rates, higher protein, vitamin C, and phenolic contents, offering a promising plant‐based frozen dessert option.

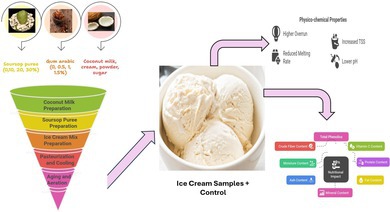

## 1 Introduction

Ice cream is a product derived from agitating and chilling a liquid blend of various components and additives. These components encompass milk products, sugar, egg products, stabilizers, colors, and flavors (Fiol et al. 2017). The amalgamation of these ingredients plays a crucial role in determining the quality of ice cream by ensuring uniformity in the final product. Furthermore, processing and storage conditions exert an impact on ice cream attributes, including overrun, pH, hardness, meltdown time, and viscosity (Silva Junior and Lannes 2011). Ice cream is characterized by its ease of digestibility and a rich array of essential nutrients (Gheisari et al. 2020). Commonly found in the market, ice creams typically consist of approximately 64% water, 18% sugars, 10% nonfat milk solids, and 8% milk solid fats (Syed et al. 2018). However, these proportions may vary based on the desired product quality and consumer preferences, such as those of lactose intolerant and vegan consumers (Szilagyi and Ishayek 2018).

The popularity of plant‐based milks, as alternatives to dairy milk, has surged in the production of ice cream and other frozen desserts. Different plant‐based milks exhibit distinct nutritional properties and physicochemical characteristics in the resultant products. A comprehensive examination by Walther et al. (2022) scrutinizes oat, rice, coconut, almond, cashew nut, and bovine milks in terms of their nutritional attributes. Coconut milk, due to its favorable physicochemical properties, has been used in formulating nondairy ice creams either independently or in conjunction with fruit pulps and herbal extracts (Góral et al. 2018; Jayasundera and Fernando 2014).

Research efforts have explored the incorporation of fruit pulps, peels, or their extracts into ice cream to enhance both physicochemical and nutritional properties (Ogo et al. 2021; Ürkek 2021; Ürkek et al. 2024; Yangılar 2015). This is because various fruits not only offer nutritional benefits but also influence the physicochemical characteristics of ice cream (Rejman et al. 2021). Soursop ( *Annona muricata* ), an evergreen plant cultivated in numerous regions worldwide, including the lowlands of Africa (Badrie and Schauss 2010; Moghadamtousi et al. 2015), is a highly perishable fruit with a short shelf life of1–3 days postripening. The fruit pulp is a rich source of carbohydrates, proteins, vitamin C, B vitamins, potassium, calcium, zinc, and phosphorus (Degnon et al. 2013). It has found applications in various food products, including juices, shakes, yoghurts, alcoholic beverages, and ice creams. Additionally, plant gums like locust bean gum, guar gum, xanthan gum, and gum arabic have been extensively employed in ice cream formulations to enhance its properties (Jagdish et al. 2015; Javidi et al. 2016; Syed and Shah 2016). Gum arabic, a complex heteropolysaccharide with a branched‐chain structure, stands out for its water solubility and relatively low viscosity in solutions and is characterized by its lack of odor, color, and aftertaste (Patel and Goyal 2015).

This study aimed to integrate soursop fruit puree and gum arabic from *Acacia senegal* var. *kerensis* into a nondairy coconut milk–based ice cream and assess their impact on the physicochemical and nutritional properties of the resultant product.

## 2 Materials and Methods

### 2.1 Raw Material Preparation

**FIGURE 1:**
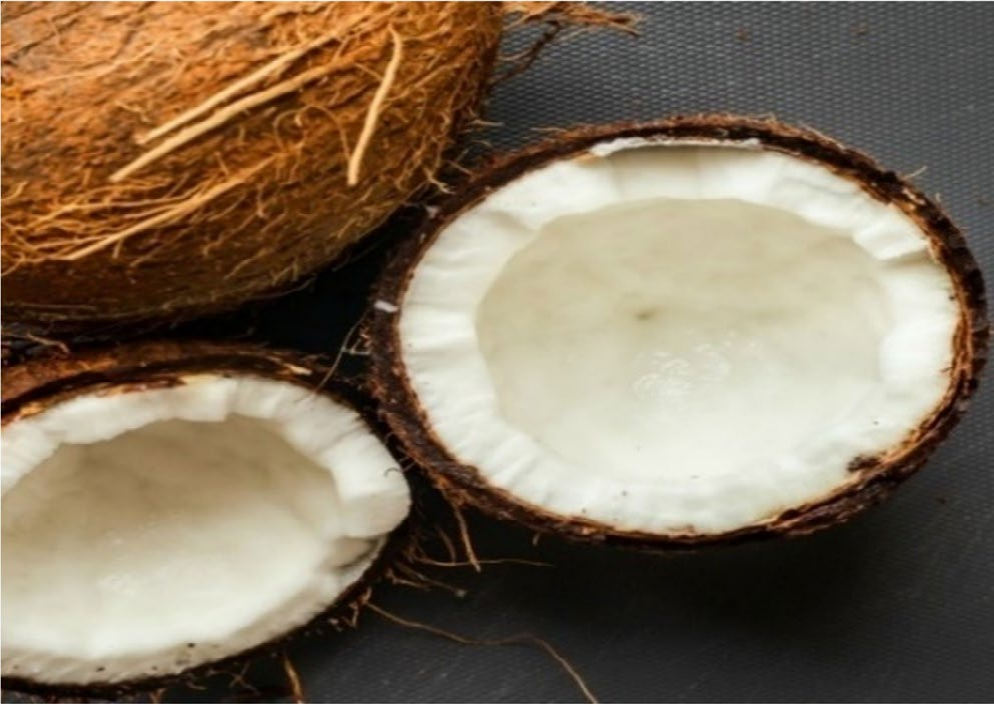
Coconut ( *Cocos nucifera* ).

**FIGURE 2:**
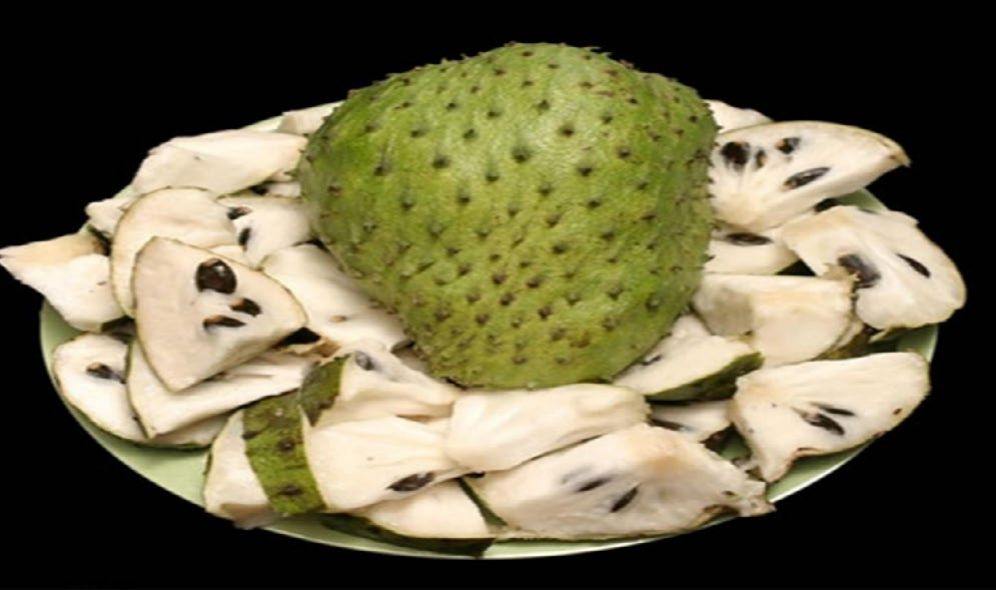
Soursop ( *Annona muricata* ) fruit.

The coconuts (Figure 1) and ripe soursop fruits (Figure 2) utilized in this study were procured from local stores in Kenya. Gum arabic was obtained from Acacia EPZ Limited, Athi River, Off Nairobi‐Namanga Highway. The raw material preparation method adhered to the procedure outlined by Perera and Perera (2021) and Virgen‐Ceceña et al. (2019) with modifications. For coconut milk preparation, the coconut fruits were halved, and the copra was extracted using a coconut scrapper. The extracted copra was then mixed with water (temperature at 70°C) in a 1:1 ratio (w/v) and blended using an electric blender. Subsequently, a clean muslin cloth was employed to extract the milk from the mixture. For soursop puree, the soursop fruits were thoroughly cleaned using running portable water, peeled, and then sliced into halves. Manual separation of seeds was carried out, and the flesh was blended to achieve a homogeneous puree.

### 2.2 Preparation of Coconut Milk–Based Ice Cream

The ice cream preparation followed the procedure outlined by Widjajaseputra and Widyastuti (2017) with some modifications. A mixture of coconut milk, coconut cream, coconut skim milk powder, fruit pulp (at concentrations of 0%, 10%, 20%, and 30%), sugar (10%), and gum arabic (at concentrations of 0%, 0.5%, 1%, and 1.5%) was combined in specified proportions to create the ice cream mix. This mix underwent pasteurization at 85°C for 30 min, followed by cooling to approximately 25°C. Subsequently, the mix was aged at 4°C for 4 h and then aerated by whipping with a Watoor ice cream machine (Model number ICM‐16A, China). The machine was programmed to operate at −18°C for 20 min per batch. Cups, precooled at 4°C, were utilized for packaging the ice cream, and the packaged ice cream was stored at −18°C in preparation for further analysis.

### 2.3 Physicochemical Property Analyses

#### 2.3.1 Analysis for pH

A pH meter (HANNA, 211; USA) was calibrated using standard buffers at pH 4.0 and 7.0. The pH of the samples was checked by dipping the pH electrode into the sample held in a 10 mL beaker according to Hashim and Shamsi (2016). The values were read directly from the display. The pH meter was rinsed using distilled water before proceeding to the next sample. The samples were run in triplicate.

#### 2.3.2 Analysis for Total Soluble Solids

A bench refractometer (ABBE 2WAJ) was used for the analysis of total soluble solids according to Rahim and Sarbon (2019). Distilled water was used to calibrate the refractometer to zero before taking the sample readings. The samples were analyzed in triplicate.

#### 2.3.3 Overrun Determination

Overrun was determined as the percentage of aeration of the dessert after freezing using the formula by Aboulfazli et al. (2015). The formula below was used to calculate the overrun.

(1) $$\text{Overrun}\;\left(\%\right)=\frac{\mathrm{wt}\;\text{of unit}\;\mathrm{mix}-\mathrm{wt}\;\text{of equal volume of}\;\mathrm{ice}\;\text{cream}}{\mathrm{Wt}\;\text{of equal volume of}\;\mathrm{ice}\;\text{cream}}\times 100$$

#### 2.3.4 Melting Rate Determination

The melting rate was determined according to the procedure described by Mahdian et al. (2012) using a 0.2 cm mesh size. Briefly, 30 g of the ice cream was placed under ambient temperatures over the mesh, and a beaker was placed below it. Melting of the ice cream was allowed to take place for 20 min, and the melted ice cream in the beaker was weighed. The amount of the melted ice cream was calculated to give the melting rate in grams/min.

### 2.4 Analysis for Nutritional Properties

#### 2.4.1 Moisture Content Determination

The oven method (Method 44‐15A; AACC 2010) was used. Two grams of each sample was accurately weighed and transferred into predried aluminium dishes. The samples were dried in a dry air oven at 105°C for 3 h and cooled in a desiccator for 10 min. The amount of moisture in percentage was calculated as the percentage weight loss during drying as follows:

(2) $$\%\text{Moisture content}=\frac{\mathrm{wt}\;\text{of}\;\mathrm{pan}+\mathrm{wet}\;\text{sample}\left(g\right)-\mathrm{wt}\;\text{of}\;\mathrm{pan}+\mathrm{dry}\;\text{sample}\left(g\right)}{\mathrm{wt}\;\text{of sample}\;\left(g\right)}\times 100$$

#### 2.4.2 Ash Content Determination

Ash content was determined using Association of Official Analytical Chemists (AOAC 2000) method 942.05. Two grams of the samples was accurately weighed and placed into silica crucibles. The analysis was done in triplicates. The samples were then ashed in a muffle furnace at 550°C for 3 h. The ash was cooled in a desiccator to room temperature and weighed. Ash content was calculated as a percentage of the dry sample.

(3) $$\%\mathrm{Ash}\;\text{content}=\frac{\mathrm{wt}\;\text{of crucible}+\mathrm{ash}\left(g\right)-\mathrm{wt}\;\text{of crucible}\left(g\right)}{\mathrm{wt}\;\text{of sample}}\times 100$$

#### 2.4.3 Crude Fat Determination

Fat analysis was done using the semicontinuous Soxhlet extraction method. The sample was first predried at 95°C–100°C under a pressure of ≤ 100 mmHg for about 5 h (Method 920.39; AOAC 2000). Two grams of the predried sample was weighed into a predried extraction thimble, with porosity permitting a rapid flow of petroleum ether. The sample was then covered with glass wool. The predried boiling flask was weighed. About 25 mL of petroleum ether was added to the extraction flask. The extraction system was set to allow extraction for 25 min. The extraction flask was dried in a hot air oven at 105°C for 30 min.

Calculation was done as follows;

(4) $$\%\mathrm{Fat}\;\text{content}=\frac{\mathrm{wt}\;\text{of flask after extraction}-\mathrm{wt}\;\text{of flask}}{\mathrm{wt}\;\text{of sample}\;}\times 100$$

#### 2.4.4 Crude Protein Determination

The determination of crude protein content in the samples followed the Kjeldahl method (Method 984.13; AOAC 2000) with slight modifications. A digestion mixture was first prepared by dissolving 2.8 g of selenium powder in 800 mL of concentrated sulfuric acid. For each sample, 2 g was accurately weighed into a Kjeldahl tube, followed by the addition of a Kjeldahl catalyst and 20 mL of the prepared digestion mixture. The resulting mixture underwent digestion at 420°C for 1 h using a Gerhardt Kjeldahl therm digester (Model: KB40; Gerhardt GMBH and CO. Kg, Germany). An additional 2.8 g of selenium powder was added to each tube, and the digestion continued at 420°C for 1.5 h. After cooling the tubes to room temperature, 50 mL of distilled water was added to each digested sample. These samples were then placed in the distillation unit, and 50 mL of 35% NaOH was added. Distillation occurred for 5 min, with the distillate collected in 50 mL of 2% boric acid using a 2200 Kjeltec TM auto distillation unit (Foss Analytical, Höganäs, Sweden). The collected distillate was titrated using 0.1 N HCl and a mixed indicator (methyl red and bromocresol green indicator in a 1:1 ratio).

The percent nitrogen obtained from the titration was multiplied by 6.25 to convert it to percent protein.

(5) $$\%N=M\;\mathrm{HCl}\times \frac{\text{Corrected acid volume}}{g\;\text{of sample}}\times \frac{14\mathrm{gN}}{\mathrm{mol}}\times 100$$

Where;

M HCl = molarity of HCl, in mol/1000 mL.

Corrected acid volume = (mL std. acid for sample) − (mL std. acid for blank).

14 = atomic weight of Nitrogen. Crude protein content was obtained by multiplying the nitrogen content by 6.25.

#### 2.4.5 Crude Fiber Determination

The determination of crude fiber content in each sample followed the AOAC (2000) Official Method 984.04. Initially, two grams of the predried sample were weighed and combined with 100 mL of hot water. Subsequently, 25 mL of 2.04 N sulfuric acid was added to the mixture, and the volume was adjusted to 200 mL. The mixture was then boiled for 30 min, with constant addition of hot water to maintain the volume at 200 mL. After the 30‐min boiling period, the mixture was filtered through glass wool and washed three times with hot water. Following this, approximately 100 mL of hot water, along with 25 mL of 1.78 N potassium hydroxide, was added. The volume was increased to 200 mL by topping up with water, and the contents were boiled for an additional 30 min. Filtration using glass wool was again performed, followed by three washes with hot water. The resulting residue was transferred into a porcelain crucible and allowed to dry in an oven set at 105°C for 2 h. After drying, the crucible was cooled in a desiccator and then weighed. The dried residue was subsequently ashed in a muffle furnace set at 550°C for 4 h. Following complete cooling in a desiccator to room temperature, the crucible was weighed once again.

(6) $$\%\text{Crude fiber}=\;\frac{\mathrm{wt}\;\text{of crucible}+\mathrm{dry}\;\text{residue}\left(g\right)-\mathrm{wt}\;\text{of crucible}+\mathrm{ash}\left(g\right)}{\mathrm{wt}\;\text{of sample}\;\left(g\right)}\times 100$$

#### 2.4.6 Mineral Analysis

Mineral analysis was conducted in accordance with the AOAC (2000) method 985.35, as outlined by Poitevin (2016) and Nielsen (2010). The quantitative determination of minerals such as Calcium (Ca), Iron (Fe), Zinc (Zn), Magnesium (Mg), Sodium (Na), and Potassium (K) was performed using a single beam atomic absorption spectrometer (AAS), with results reported in parts per million (ppm) (1 ppm = 1 mg/kg). Calibration curves for each element were established using working standards. For sample preparation, 1 g of the dried sample was combined with 5 mL of concentrated sulfuric acid (H_2_SO_4_) in a flask and heated on a hot plate at 200°C until brown–yellow fumes appeared, indicating the decomposition of organic matter. Once the brown–yellow fumes ceased and white fumes emerged from decomposing H_2_SO_4_, the flask was removed from the heat. Gradually, 3–5 mL of nitric acid (HNO_3_) was added to the flask before it cooled to room temperature, and the flask was reheated until all HNO_3_ evaporated, leaving a clear to straw‐yellow solution. This process was repeated by adding further increments of HNO_3_ until the solution consistently achieved a straw‐yellow color with a target volume reduction to around 10–15 mL for easier transfer into volumetric flasks. Upon cooling, the digested samples were quantitatively transferred to 50 mL volumetric flasks and diluted with distilled water for the specific minerals being analyzed. Throughout the analysis, control samples and replicates were included, and the AAS was calibrated according to the instrument's specifications for each mineral being measured. The ashed samples were then transferred to AAS auto sampler vials for the analysis of Calcium (Ca), Iron (Fe), Sodium (Na), Magnesium (Mg), Zinc (Zn), and Potassium (K) following the procedure outlined by Okon and Ojimelukwe (2017).

#### 2.4.7 Vitamin C Determination

Vitamin C was determined using AOAC Method 967.21, 45.1.14 (Nielsen 2010). Two grams of the sample was weighed accurately and homogenized in metaphoric acid and acetic acid solution (15 g HPO_4_ and 40 mL CH_3_COOH topped up with water to 500 mL). The sample extract was then filtered and diluted to a final concentration of between10 and 100 mg ascorbic acid/100 mL.

A standard solution was prepared by dissolving 50 mg of L‐ascorbic acid in 100 mL of water. In triplicates, titration of the samples was done with the standard solution with 2,6‐dichloroindophenol solution to a pink endpoint lasting at least 10 s. Results were recorded for calculation as follows:

(7) $$\mathrm{Mg}\;\text{Ascorbic acid}\;\mathrm{per}\;\text{gram}=C\times V\times \frac{\mathrm{DF}}{\mathrm{Wt}}$$

where: C = mg ascorbic acid

V = mL dye used for titration of diluted sample

DF = dilution factor

Wt = sample weight, g

#### 2.4.8 Determination of Total Phenolic Content

The total phenolic content of the samples was assessed utilizing the Folin–Ciocalteu method, as outlined by Ullah et al. (2017). A 0.1 mg portion of the ice cream sample was mixed with 5.9 mL of deionized water. Subsequently, 1 mL of the diluted ice cream sample was combined with 1 mL of Folin–Ciocalteu reagent. After allowing the mixture to stand for 5 min, 2 mL of 20% (w/v) sodium carbonate was added. The contents of the test tube were stirred and then incubated at room temperature for 10 min, followed by centrifugation at 1500× *g*. Absorbance was measured using a double‐beam spectrophotometer at 550 nm. The determination of total phenolic content was based on a calibration curve using Gallic acid as a standard (ranging from 10 to 100 ppm) and reported as Gallic Acid Equivalent (GAE) in milligrams per gram (mg/100 g).

### 2.5 Statistical Data Analysis

The experiment utilized a completely randomized design (CRD) in a 4 × 4 factorial arrangement. The first factor consisted of varying percentages of soursop puree at four levels: 0%, 10%, 20%, and 30%, while the second factor involved different levels of gum arabic at 0%, 0.5%, 1.0%, and 1.5%. The control group had 0% incorporation of both factors, with a conventional stabilizer and emulsifier. Data obtained from the experiment were analyzed using Statistical Analysis System (SAS) software Version 9.1.3. To test study hypotheses, analysis of variance (ANOVA) was conducted. Significance was established at a confidence level of *p* < 0.05, and means were separated using the Duncan's Multiple Range Test (DMRT) method.

## 3 Results and Discussion

### 3.1 Effect of Soursop Puree and Gum Arabic Incorporation and Their Interaction on the Physicochemical Properties of Nondairy Coconut Milk–Based Ice Cream

Physicochemical properties such as overrun, melting rate, pH, and total soluble solids are important for evaluating the texture, stability, and flavor of ice cream, each affecting its quality in different ways. Table 1 summarizes the impact of different levels of soursop puree and gum arabic on these properties in nondairy, coconut milk–based ice cream. Additionally, Table 2 shows the interaction effects between soursop puree and gum arabic, highlighting how their combinations influence the physicochemical properties to improve product quality.

**TABLE 1:** Physicochemical properties of ice cream influenced by different levels of soursop puree and gum arabic incorporation.

Levels (%)	Overrun (%)	Melting Rate (g/min)	pH	Total soluble solids (°Bx)
*Soursop puree*				
0	14.90 ± 1.05ᶜ	0.97 ± 0.04ᵃ	6.15 ± 0.03ᵃ	34.90 ± 0.35ᵃ
10	16.32 ± 1.55ᶜ	0.15 ± 0.01ᵇ	5.08 ± 0.02ᵇ	31.96 ± 0.26ᶜ
20	20.01 ± 1.14ᵇ	0.10 ± 0.02ᵇ	4.61 ± 0.03ᶜ	33.35 ± 0.42ᵇ
30	26.99 ± 1.41ᵃ	0.09 ± 0.01ᵇ	4.39 ± 0.04ᵈ	32.46 ± 0.50ᶜ
*Gum arabic*				
0	18.27 ± 1.20ᵃ	0.31 ± 0.10ᵇ	5.04 ± 0.23ᵇ	32.08 ± 0.36ᶜ
0.5	18.41 ± 2.29ᵃ	0.38 ± 0.13ᵃ	5.18 ± 0.18ᵃ	32.94 ± 0.59ᵇ
1	20.50 ± 2.36ᵃ	0.32 ± 0.12ᵇ	5.00 ± 0.21ᶜ	33.81 ± 0.35ᵃ
1.5	21.04 ± 1.41ᵃ	0.30 ± 0.11ᵇ	5.00 ± 0.20ᶜ	33.83 ± 0.53ᵃ

*Note:* Values are mean scores ± standard error (*n* = 3). Means followed by different superscript letters in the same column for both soursop puree and gum arabic levels are significantly different (*p* < 0.05).

**TABLE 2:** Physicochemical properties of ice cream due to the interaction between different levels of soursop puree and gum arabic.

Soursop (%)	Gum (%)	Overrun (%)	Melting Rate (g/min)	pH	Total soluble solids (°Bx)
0	0 (Control)	14.10 ± 0.64ᵈ	0.87 ± 0.06^b^	6.28 ± 0.01ᵃ	33.67 ± 0.30ᵈᵉᶠ
	0.5	12.35 ± 1.63ᵈ	1.12 ± 0.10ᵃ	6.18 ± 0.01ᵇ	34.33 ± 0.82ᵇᶜᵈᵉ
	1	15.09 ± 2.88ᵈ	0.98 ± 0.04ᵇ	6.12 ± 0.01ᶜ	35.50 ± 0.29ᵃᵇ
	1.5	18.06 ± 2.05ᵇᶜᵈ	0.90 ± 0.08ᵇ	6.02 ± 0.01ᵈ	36.08 ± 0.22ᵃ
10	0	16.67 ± 1.28ᶜᵈ	0.17 ± 0.02ᶜ	5.08 ± 0.00ᶠ	31.08 ± 0.30^ij^
	0.5	12.78 ± 1.47ᵈ	0.15 ± 0.02ᶜ	5.16 ± 0.00ᵉ	31.50 ± 0.14^hij^
	1	12.58 ± 0.63ᵈ	0.11 ± 0.01ᶜ	4.99 ± 0.00ᵍ	33.08 ± 0.22ᵉᶠᵍ
	1.5	23.27 ± 0.33ᵃᵇᶜ	0.16 ± 0.02ᶜ	5.07 ± 0.00ᶠ	32.17 ± 0.44ᵍʰ^i^
20	0	18.24 ± 1.26ᵇᶜᵈ	0.08 ± 0.01ᶜ	4.49 ± 0.01ʲ	32.00 ± 0.29^ghij^
	0.5	18.95 ± 3.46ᵇᶜᵈ	0.15 ± 0.07ᶜ	4.78 ± 0.04ʰ	35.17 ± 0.22ᵃᵇᶜ
	1	24.18 ± 1.31ᵃᵇ	0.09 ± 0.01ᶜ	4.59 ± 0.01^i^	33.92 ± 0.30ᶜᵈᵉᶠ
	1.5	18.67 ± 1.33ᵇᶜᵈ	0.09 ± 0.01ᶜ	4.58 ± 0.03^i^	32.33 ± 0.65^ghij^
30	0	24.07 ± 1.07ᵃᵇ	0.11 ± 0.02ᶜ	4.33 ± 0.04ᵏ	31.58 ± 0.79^hij^
	0.5	29.56 ± 1.26ᵃ	0.11 ± 0.01ᶜ	4.61 ± 0.02^i^	30.75 ± 0.29^j^
	1	30.13 ± 3.57ᵃ	0.08 ± 0.01ᶜ	4.29 ± 0.03ᵏ	32.75 ± 0.43ᶠᵍʰ
	1.5	24.18 ± 3.46ᵃᵇ	0.07 ± 0.01ᶜ	4.33 ± 0.04ᵏ	34.75 ± 0.29ᵇᶜᵈᵉ

*Note:* Values are means ± standard error (*n* = 3). Means followed by different superscript letters in the same column are significantly different (*p* < 0.05).

#### 3.1.1 Overrun

Overrun, denoting the expansion in volume of frozen desserts due to the incorporation of air, is expressed as a percentage (VanWees et al. 2020). While overrun varies based on the desired final product, ingredient types, and processing methods, its determination is crucial as it serves as a quality indicator. Ice cream incorporated with different levels of soursop puree and gum arabic reveals differences in overrun (Table 1), demonstrating how these ingredients impact texture and air content. Ice cream formulations containing 20% and 30% soursop puree exhibited significantly (*p* < 0.05) elevated overrun values (20.01% ± 1.14% and 26.99% ± 1.41%, respectively) compared to 0% (14.90% ± 1.05%) and 10% (16.32% ± 1.55%) incorporation levels. Similarly, ice cream samples incorporating 1% and 1.5% gum arabic displayed increased overrun values (20.50% ± 2.36% and 21.04% ± 1.41%, respectively) compared to those with 0% and 0.5% gum arabic (18.27% ± 1.20% and 18.41% ± 2.29% respectively). There was a direct relationship between the levels of soursop puree and gum arabic added and the overrun in the ice cream samples. However, no statistically significant difference (*p* > 0.05) was noted in terms of overrun among ice cream samples with varying levels of gum arabic. The interaction effect (Table 2) between gum arabic and soursop puree proved to be significant (*p* < 0.05) for overrun at different levels of incorporation. Treatments with higher combinations of soursop and gum displayed the highest overrun, along with the lowest pH and melting rate. Overrun increased proportionally with higher levels of substitution for both soursop puree and gum arabic. The highest overrun values were observed at 20% and 30% soursop levels, showing an increase with rising levels of gum arabic up to 1%, and then a decline at 1.5%. However, the impact on overrun due to interaction did not differ significantly among treatments with the same soursop puree levels (*p* > 0.05). The decrease in overrun resulting from gum incorporation above 1% can be attributed to the increased viscosity of the mix, making it challenging to introduce air during whipping (Syed et al. 2018). A lower overrun leads to a firmer frozen dessert with faster meltability, while a higher overrun gives an easier‐to‐scoop product with a slower meltability, which is preferred by consumers (Aboulfazli et al. 2015). Hydrocolloid polysaccharides, such as gum arabic, contribute to foam stability by increasing mix viscosity, thereby impeding water phase separation (Sikora et al. 2008). This improved foam stability results in more trapped air during whipping, leading to a higher overrun (Pasban et al. 2014). The high overrun with increasing levels of gum arabic in this study aligns with the report by Yudhistira et al. (2018), where the combination of gum arabic and carboxymethyl cellulose (CMC) in a frozen dessert made from jackfruit exhibited the highest overrun of 25.015%.

#### 3.1.2 pH

The acidity of ice cream plays a crucial role in determining flavor, texture, and overall quality (Syed et al. 2018). Tables 1 and 2 further illustrate the effects of soursop puree and gum arabic addition on the pH of ice cream. Increasing the levels of soursop puree in the ice cream mix resulted in a significant increase in acidity, consequently causing a significant reduction in the pH of the ice cream (*p* < 0.05). The addition of soursop puree at 20% and 30% (Table 1) led to the lowest pH values (4.61 ± 0.03 and 4.39 ± 0.04, respectively). Higher levels of gum arabic substitution (1% and 1.5%) did not exert a significant impact (*p* > 0.05) on the pH of the ice cream (Table 1). The interaction between soursop and gum arabic (Table 2) resulted in low pH values ranging from 4.29 to 5.16, while treatments without soursop exhibited higher pH values ranging from 6.02 to 6.28. Despite displaying neutral characteristics, gum arabic, when combined with slightly acidic soursop, led to the lowest pH values in treatments with 30% soursop levels. According to Al‐Assaf et al. (2009), gum arabic dissolves in water, yielding a clear solution with a pH of approximately 4.5. pH is a critical aspect in ice cream quality as it influences the structure of the ice cream. At lower pH values, the proteins in the ice cream undergo denaturation, causing them to adopt an extended formation that impedes the ice cream from dripping through the screen. Ice cream with lower pH values tends to maintain its structure longer after scooping compared to those with higher pH values. Therefore, this could explain the noted observation. Similarly, Fávaro‐Trindade et al. (2007) reported that ice cream samples with a pH of 4.5 exhibited lower melting rates than those with a pH value of 5. In a study by Öztürk‐Yalçın et al. (2024), ice cream samples fermented using kefir culture exhibited pH values ranging from 4.65 to 4.98. The pH results in the present study, particularly after the addition of soursop puree, align with these findings. While fermentation is known to lower pH, the incorporation of soursop puree similarly contributed to a pH reduction. The characteristic acidity of soursop puree intensifies during ripening, with ripe soursop fruit pulp exhibiting a pH range between 4.1 and 4.8 (Degnon et al. 2013). The acidity in soursop fruit pulp is attributed to the presence of malic and citric acid, as indicated by Bhat and Paliyath (2016), explaining the observed low pH values at higher soursop addition levels.

#### 3.1.3 Melting Rate

Melting rate, reflecting the time required for ice cream to melt, is vital in assessing ice cream stability under ambient temperatures (Mahdian et al. 2012). Ice cream samples containing 0% soursop puree displayed the highest rate (0.97 ± 0.04 g/min), while those with 30% soursop exhibited the lowest rate (0.09 ± 0.01 g/min), as shown in Table 1. Similarly, the addition of 1.5% gum arabic displayed the lowest melting rate (0.30 ± 0.11 g/min), although the difference was not significant (*p* > 0.05) in samples containing 1% gum arabic (0.32 ± 0.12 g/min) compared to 1.5%. The highest melting rate (0.38 ± 0.13 g/min) was observed with 0.5% gum arabic addition. Melting rate decreased with increased levels of soursop puree and gum arabic addition. A significant difference was noted between samples containing soursop puree and those without (*p* < 0.05). There was no significant difference in the effect of melting rate due to interaction (Table 2) in treatments containing 10%, 20%, and 30% soursop levels (*p* > 0.05). However, samples without soursop exhibited a significant difference in terms of melting rate compared to the rest (*p* < 0.05). The starchy composition of soursop fruit puree likely contributes to the mix's viscosity, thereby lowering the melting rate. Góral et al. (2018) reported that coconut milk, rich in polysaccharides, requires higher stabilizer quantities to increase the meltdown time. In their study, ice cream mixes with increased locust bean gum content did not melt easily. Gum arabic, having been used as a stabilizer in low‐fat yogurt (Mugo et al. 2020), presents a potential application as a stabilizer in ice cream mixes to reduce the melting rate. Higher melting rates are associated with lower overrun, while lower melting rates are linked to higher overrun. This relationship is attributed to the fact that higher overrun, characterized by increased air content, slows down heat transfer in the ice cream (Muse and Hartel 2004). Consistent with Yudhistira et al. (2018), melting rate reduced with increased levels of gum arabic when used in combination with carboxymethyl cellulose (CMC) in a frozen dessert made from jackfruit.

#### 3.1.4 Total Soluble Solids (TSS)

Total Soluble Solids play an important role in determining the texture of ice cream. Samples containing 1.5% gum arabic exhibited the highest concentration of TSS (33.83° ± 0.53°Bx), while those with 0% gum arabic had the lowest (32.08° ± 0.36°Bx). A significant difference was observed between samples with varying levels of gum arabic and those without (*p* < 0.05). The concentration of TSS demonstrated an inverse relationship with the levels of gum arabic in the ice cream. The TSS content is influenced by the quantity and nature of ingredients in the ice cream mix formulation, with the addition of sweeteners like sugar and honey contributing to increased TSS. Rahim and Sarbon (2019) reported lower TSS values in ice cream samples with the addition of different hydrocolloids to an acacia honey lime mix. In their study, guar gum yielded a TSS of 30.77° ± 0.51°Bx, xanthan gum (31.43° ± 1.72°Bx), and carboxymethyl cellulose (29.27° ± 2.83°Bx), values lower than those obtained in this study. Gum arabic dissolves in water to produce polyelectrolyte solutions of low viscosity that decrease further under acidic conditions. The decrease is due to the presence of carboxyl groups (Al‐Assaf et al. 2009). Mix viscosity is a critical factor in ice cream mix formulation as it influences ice cream overrun. High viscosity in the mix is desirable to increase overrun until an optimum level (TSS of 40%–42%), where whip ability decreases, resulting in decreased overrun (Syed et al. 2018).

### 3.2 Correlation Coefficients of the Physicochemical Properties of the Nondairy Coconut Milk–Based Ice Cream

Testing for correlation is important in understanding the relationships between ice cream properties, such as overrun, melting rate, total soluble solids, and pH. The correlation coefficients for the different physicochemical properties are presented in Table 3. A statistically significant (*p* < 0.01) negative correlation (−0.43) was observed between overrun and melting rate (Table 3). This indicates that as the quantity of air incorporated during whipping increases, the ice cream exhibits a tendency to resist melting. The presence of air in ice cream serves as an insulator, prolonging the time required for the ice cream to fully melt. The weak negative correlation may be influenced by other contributing factors affecting both the reduction in melting rate and the increase in overrun (Warren and Hartel 2018). As highlighted by Liu et al. (2023), the type and amounts of emulsifiers used can impact the melting properties and overrun. Different emulsifiers have varying effects on fat emulsions, causing protein displacement from the interface and resulting in a thinner interface that favors coalescence during freezing. The results further revealed a strong positive correlation (0.92) between melting rate and pH, significant at *p* < 0.001 (Table 3). This suggests that the melting rate increases with higher pH values of the ice cream and decreases as the pH decreases. A lower pH signifies a more acidic environment, favoring protein denaturation, as presented by Fávaro‐Trindade et al. (2007). Protein denaturation leads to an irreversible elongation of the protein structure, making it challenging for the ice cream to drip through the mesh. This observation aligns with the significant negative correlation (−0.59) between overrun and pH at *p* < 0.001 (Table 3). Higher overrun is associated with a lower melting rate of ice cream, where the amount of air incorporated during manufacturing plays a crucial role. According to Sakurai (1996), ice cream with high overrun tends to melt more slowly than those with lower overruns, primarily due to the higher volumes of air resulting in reduced heat transfer (Hartel et al. 2004).

**TABLE 3:** Correlation coefficients for the different physicochemical properties of ice cream.

	Overrun (%)	Melting rate (g/min)	pH	Total soluble solids (°Bx)
Overrun (%)	1	−0.43**	−0.59***	−0.23^ns^
Melting Rate (g/min)		1	0.92***	0.54***
pH			1	0.46**
Total soluble solids (°Bx)				1

*Note:* ***Significant at *p* < 0.001, **Significant at *p* < 0.01, ns Not significant at *p* < 0.05.

### 3.3 Effect of Soursop Puree and Gum Arabic Incorporation on the Proximate and Total Phenolic Composition of Nondairy Coconut Milk–Based Ice Cream

Analyzing the proximate and total phenolic compositions of ice cream is essential for assessing its quality and possible health benefits. Important factors include moisture, ash, protein, total phenolics, vitamin C, fat, and fiber contents, which collectively determine the nutritional value of nondairy ice cream. The impact of adding soursop puree and gum arabic on these nutritional aspects is summarized in Table 4. Furthermore, the interaction of these ingredients has a combined impact on the overall nutritional profile, and Table 5 highlights these effects.

**TABLE 4:** Proximate and total phenolic composition of ice cream incorporated with soursop puree and gum arabic.

Levels (%)	Protein (%)	Fat (%)	Ash (%)	Fiber (%)	MC (%)	Vitamin C (mg/100 g)	Phenolics (mg GAE/100 g)
*Soursop*							
0	4.39 ± 1.69ᵈ	24.76 ± 6.44ᵃ	3.55 ± 0.89ᵇ	0.37 ± 0.29ᶜ	53.30 ± 2.08ᶜ	5.36 ± 0.23ᵈ	408.11 ± 36.13ᵈ
10	7.10 ± 2.31ᵇ	15.47 ± 3.20ᵇ	2.95 ± 1.59ᶜ	0.78 ± 0.53ᶜ	55.29 ± 3.09ᵇ	7.10 ± 2.89ᶜ	1457 ± 29.73ᶜ
20	5.11 ± 3.15ᶜ	23.82 ± 3.97ᵃ	4.41 ± 2.06ᵃ	11.23 ± 5.18ᵇ	56.75 ± 3.54ᵇ	9.31 ± 4.22ᵇ	1748.54 ± 29.85ᵇ
30	9.50 ± 4.06ᵃ	14.13 ± 5.53ᵇ	2.42 ± 0.72ᵈ	21.87 ± 7.03ᵃ	64.96 ± 3.72ᵃ	22.66 ± 5.78ᵃ	4332.53 ± 48.36ᵃ
*Gum*							
0	5.42 ± 3.03ᶜ	21.15 ± 7.16ᵃ	3.14 ± 1.59ᵇᶜ	6.29 ± 8.11ᶜ	57.39 ± 4.39ᵃᵇ	10.17 ± 8.40ᵇ	1991.27 ± 1526.29ᵃᵇ
0.5	6.23 ± 3.50ᵇ	20.13 ± 9.34ᵃ	3.90 ± 1.82ᵃ	11.42 ± 13.36ᵃ	59.04 ± 7.04ᵃ	12.63 ± 9.57ᵃ	1987.49 ± 1502.37ᵃᵇ
1	8.55 ± 3.65ᵃ	20.43 ± 6.35ᵃ	3.32 ± 1.45ᵇ	8.01 ± 8.90ᵇᶜ	56.24 ± 5.18ᵇ	9.43 ± 4.55ᵇ	1967.89 ± 1498.35ᵇ
1.5	5.90 ± 3.27ᵇᶜ	16.47 ± 2.11ᵇ	2.97 ± 1.41ᶜ	8.51 ± 8.86ᵇ	57.64 ± 5.11ᵃᵇ	12.20 ± 8.45ᵃ	1999.71 ± 1504.18ᵃ

*Note:* Values are mean scores ± standard error (*n* = 3). Means followed by different superscript letters in the same column are significantly different (*p* < 0.05).

Abbreviations: GAE, gallic acid equivalent; MC, moisture content.

**TABLE 5:** Proximate and total phenolic composition of ice cream due to interaction between soursop puree and gum arabic.

Soursop (%)	Gum (%)	Protein (%)	Fat (%)	Ash (%)	Fiber (%)	MC (%)	Vit. C (mg/100 g)	Phenolics (mg GAE/100 g)
0	0	6.97 ± 0.52ᵈ	28.41 ± 1.83ᵃ	2.19 ± 0.16ᵉ	0.28 ± 0.16ᵉ	54.47 ± 1.37ᶠᵍ	5.49 ± 0.16ᵈ	420.88 ± 5.88ᶠ
	0.5	4.28 ± 0.38ᵉᶠ	30.20 ± 2.88ᵃ	4.15 ± 0.02ᶜ	0.63 ± 0.48ᵉ	53.29 ± 0.92ᵍ	5.35 ± 0.10ᵈ	409.95 ± 18.16ᶠ
	1	2.80° ± 0.34°ᵍ	25.57 ± 1.49ᵇ	3.58 ± 0.49ᵈ	0.23 ± 0.15ᵉ	50.57 ± 1.42ᵏ	5.06 ± 0.15ᵈ	361.36 ± 31.11^g^
	1.5	3.50 ± 0.53ᶠᵍ	14.85 ± 1.30ᵉ	4.26 ± 0.24ᶜ	0.32 ± 0.19ᵉ	54.88 ± 1.43ᵉᵖ	5.54 ± 0.18ᵈ	440.24 ± 27.77ᶠ
10	0	9.29 ± 1.06ᶜ	17.67 ± 1.56ᵈ	3.38 ± 0.24ᵈ	1.30 ± 0.41ᵉ	58.19 ± 2.09ᵈᵉ	5.99 ± 0.29ᵈ	1436.55 ± 21.28ᵉ
	0.5	4.92 ± 0.50ᵉ	18.32 ± 1.29ᵈ	2.22 ± 0.22ᵉ	0.27 ± 0.08ᵉ	57.73 ± 1.65ᵈᵉ	11.84 ± 0.46ᶜ	1446.31 ± 36.60ᵉ
	1	9.23 ± 0.40ᶜ	11.04 ± 0.95ᶜ	5.15 ± 0.15ᵇ	1.14 ± 0.37ᵉ	52.75 ± 1.14ᵍ	5.29 ± 0.13ᵈ	1481.95 ± 14.19ᵉ
	1.5	4.97 ± 0.11ᵉ	14.84 ± 1.32ᵉ	1.05 ± 0.04ᶠ	0.40 ± 0.09ᵉ	52.48 ± 1.08ᵍ	5.26 ± 0.12ᵈ	1463.88 ± 32.65ᵉ
20	0	2.38 ± 0.43ᵏ	26.71 ± 0.61ᵇ	5.48 ± 0.25ᵇ	4.09 ± 0.32ᵉ	53.39 ± 1.10ᵍ	5.20 ± 0.17ᵈ	1729.25 ± 19.21ᵈ
	0.5	4.01 ± 0.10ᵉᶠ	25.21 ± 2.78ᶜ	6.61 ± 0.28ᵃ	13.84 ± 2.54ᵗ	55.42 ± 1.13ᵉᵍ	5.61 ± 0.14ᵈ	1772.37 ± 25.84ᵈ
	1	10.12 ± 0.97ᵇ	25.36 ± 2.63ᶜ	1.35 ± 0.29ᶠ	10.05 ± 0.66ᵈ	58.72 ± 1.20ᵈ	13.47 ± 2.15ᶜ	1745.11 ± 41.03ᵈ
	1.5	3.95 ± 0.87ᵉᶠ	18.01 ± 1.38ᵈ	4.19 ± 0.22ᶜ	16.94 ± 1.67ᵇ	59.47 ± 5.31ᶜ	12.97 ± 1.07ᶜ	1747.45 ± 27.42ᵈ
30	0	3.05 ± 0.63ᶠᵍ	11.79 ± 0.91ᶜ	1.49 ± 0.22ᶠ	19.50 ± 0.96ᵇ	63.51 ± 2.18ᵇ	23.99 ± 2.33ᵇ	4378.40 ± 18.61ᵃ
	0.5	11.72 ± 2.50ᵃ	6.80 ± 0.53ᵍ	2.63 ± 0.52ᵉ	30.93 ± 5.86ᵃ	69.71 ± 5.00ᵃ	27.71 ± 2.83ᵃ	4321.33 ± 4.23ᵇ
	1	12.04 ± 0.15ᵃ	19.77 ± 1.66ᵈ	3.18 ± 0.52ᵈ	20.64 ± 5.29ᵇ	62.91 ± 0.47ᵇ	13.89 ± 0.78ᶜ	4283.14 ± 65.58ᶜ
	1.5	11.19 ± 0.24ᵃ	18.18 ± 1.80ᵈ	2.39 ± 0.24ᵉ	16.39 ± 5.44ᵇ	63.72 ± 0.69ᵇ	25.04 ± 2.18ᵇ	4347.26 ± 29.53ᵃ

*Note:* Values are mean scores ± standard error (*n* = 3). Means followed by different superscript letters in the same column are significantly different (*p* < 0.05).

Abbreviations: Carbs, carbohydrates; GAE, gallic acid equivalent; MC, moisture content; Vit. C, vitamin C.

#### 3.3.1 Protein Content

Determining the protein content in ice cream is crucial, as it influences viscosity, foaming properties, and overrun, all of which collectively impact the texture of the final product. The impact on nutritional composition resulting from the incorporation of soursop puree and gum arabic in coconut milk–based ice cream is summarized in Tables 4 and 5 above. For the different levels of soursop incorporation, the highest protein content (9.50% ± 4.06%) was observed in samples containing 30% soursop, while those without soursop exhibited the lowest protein content (4.39% ± 1.69%) as shown in Table 4. In the case of gum arabic incorporation, 1% demonstrated the highest protein content (8.55% ± 3.65%), with 0% having the lowest (5.42% ± 3.03%) (Table 4). The interaction between soursop puree and gum arabic (Table 5) yielded a protein content range of (2.38% ± 0.43%) to (12.04% ± 0.15%). The control sample, devoid of both gum arabic and soursop puree, displayed a protein content of (6.97% ± 0.52%). These findings suggest a higher protein content in the ice cream compared to the results obtained by other researchers. In the study by Beegum et al. (2022), a lower protein content range of (3.42% ± 0.11%) to (4.94% ± 0.22%) was reported after substituting traditional ice cream ingredients, such as butter, skim milk powder, sugar, and dairy milk, with coconut‐based alternatives like coconut milk, coconut sugar, tender coconut pulp, and coconut water. Similarly, Perera and Perera (2021) found a protein content of (4.18% ± 0.16%) in a spicy coconut milk–based ice cream. Beegum et al. (2022) indicated that the average protein content in coconut milk is approximately (6.79% ± 0.05%). The elevated protein levels in this study are attributed to the ice cream formulation incorporating coconut milk, coconut cream, and coconut skim milk powder. Proteins play a crucial role in ice cream manufacturing due to their emulsification, whip ability, and foaming properties (Roy et al. 2022). Essential to ice cream quality are foaming capacity and foam stability. Foaming capacity involves proteins creating an interfacial area in the liquid phase during whipping (Patel and Kilara 1990), while foam stability describes the time needed to expel 50% of the volume in the foam (Ivanova et al. 2018). These functional properties impact overrun, contributing to a smooth and consistent body and texture in the resulting ice cream (Legassa 2020). Afzaal et al. (2022) reported that the pulp of a ripe, healthy soursop fruit contains about 1.20%–1.24% crude protein content, while Kauther and Hussien (2018) reported a protein content of 2.2% in gum arabic. This suggests that the observed trend in protein content is primarily influenced by the base ingredients rather than the incorporation of soursop puree and gum arabic.

#### 3.3.2 Fat Content

Fat content in ice cream impacts flavor and texture of the resulting product. The samples with 0% soursop and 0% gum arabic recorded the highest fat content (Table 4). As the level of soursop puree addition increased up to 30%, there was a corresponding decrease (14.13% ± 5.53%) in the fat content of the subsequent ice cream samples. Similarly, the addition of gum arabic up to 1.5% also led to a decrease (16.47% ± 2.11%) in the fat content of the ice cream. The interaction effect between soursop puree and gum arabic at 30% and 0.5%, respectively, resulted in the lowest (6.80% ± 0.53%) fat content in the ice cream samples, while the highest (30.20% ± 2.88%) fat content was obtained at 0% soursop puree and 0.5% gum arabic (Table 5). These findings align with those of Winarsih et al. (2020), who formulated ice cream using coconut milk and kidney nut puree, resulting in fat content ranging between 17.31% and 25.32%. Standard, high‐quality ice cream typically contains a minimum of 10%–12% fat. Pure coconut milk, without added water, comprises 33.4% fat, while coconut milk cream, a key ice cream ingredient, contains 35.34% fat, as indicated by Jayasundera and Fernando (2014). The addition of water during coconut milk extraction results in 5.8% fat in the milk, with 50.1% of these fats being predominantly lauric fatty acids (Nadeeshani et al. 2015). Soursop, being a low‐fat fruit with an average fat content of 0.3%–0.7%, had a minimal impact on the fat content of the ice cream (Inkalaba et al. 2023). Thus, the primary contributors to the fat content in the ice cream were the base coconut ingredients, not the soursop puree or gum arabic. As emphasized by Méndez‐Velasco and Goff (2012), fat plays a crucial role in ice cream manufacturing, contributing to the structural and sensory qualities of the final product. High fat content in ice cream is desirable as it enhances viscosity, aiding in retaining the structure of the ice cream by reducing its melting rate. Practical fat reduction involves increasing the aqueous phase of the ice cream mix, with bulking agents added to compensate for the viscosity reduction (Rolon et al. 2017).

#### 3.3.3 Ash Content

Determining ash content serves as the initial step in assessing the mineral composition of food products. The ash content exhibited a range of (2.42% ± 0.72%) to (4.41% ± 2.06%) for the individual effects of soursop addition and (2.97% ± 1.41%) to (3.90% ± 1.82%) for the effect resulting from the addition of gum arabic (Table 4). In the case of the interaction effect (Table 5), the ash content varied between (1.05% ± 0.04%) and (6.61% ± 0.28%). The influence of different levels of soursop puree on the ash content was found to be significantly different (*p* < 0.05). Similarly, the impact of gum arabic incorporation on the ash content showed significant differences for 0.5%, 1%, and 1.5% levels of gum arabic (*p* < 0.05). These findings concur with those reported by Ahmed and El Zubeir (2015). These researchers formulated ice cream using camel milk, incorporating honey and gum arabic, and flavored with vanilla and coconut. The ice cream thus formulated resulted in an ash content ranging from 3.42% ± 0.22% to 4.00% ± 0.28%. However, in another study by Yangılar (2015), ice cream enriched with date fiber showed a lower ash content ranging from 0.92% ± 0.01% to 1.06% ± 0.02%, compared to 0.89% ± 0.01% for the control treatment without any incorporated date fiber. Ash content is indicative of the inorganic matter present in food and is commonly used qualitatively to signify the mineral content in a food product (Harris and Marshall 2017).

#### 3.3.4 Fiber Content

Dietary fiber plays a crucial role in improving digestion and reducing the risks of obesity and cardiovascular diseases. The fiber content of the ice cream samples exhibited an increase for every 10% unit increase in the incorporation of soursop puree, ranging from the lowest at (0.37% ± 0.29%) to the highest at (21.87% ± 7.03%). The rise in fiber content due to the increased levels of soursop puree was found to be significantly different (*p* < 0.05). Ice cream samples without gum arabic had the lowest fiber content (6.29% ± 8.11%), while those with 0.5% gum arabic had the highest fiber content (11.42% ± 13.36%), as shown in Table 4. At 0% and 10% soursop puree incorporation levels, the interaction effect within the same level of soursop puree was not significantly different (*p* > 0.05) for fiber content (Table 5). However, at higher levels of soursop puree (20% and 30% substitution), there was an increase in fiber content due to the interaction effect between soursop puree and gum arabic. The highest fiber content (30.93% ± 5.86%) resulted from the interaction between 30% soursop puree and 0.5% gum arabic, while the lowest (0.23% ± 0.15%) was observed for the interaction between 0% soursop puree and 1.5% gum arabic. The results obtained from this study were higher than those reported in other studies. In a study where ice cream was enriched using bacterial cellulose and inulin to enhance dietary fiber content, the reported fiber content was 2.39% (Xavier and Ramana 2022). In the context of ice cream manufacturing, dietary fiber contributes to enhanced texture and water holding capacity due to its ability to form gels in solutions (Crizel et al. 2014). Salehi (2021) has highlighted that incorporating fruits into dairy products increases the fiber content. Osei et al. (2023) reported that ripe soursop pulp contains 6.5% crude fiber, while Tulashie et al. (2022) noted an average fiber content of 2.2% in coconut milk. Additionally, Beegum et al. (2021) found a fiber content of 22.03% in coconut milk residue. These fiber levels in soursop and coconut milk are somewhat lower than those observed in this study's ice cream, suggesting that the high fiber content here is likely due to the combined effect of all ingredients rather than solely the addition of soursop puree. Furthermore, traces of coconut milk residue may have been introduced during extraction, potentially contributing to the elevated fiber content. The addition of soursop to frozen desserts has been shown to elevate the fiber content, while frozen desserts incorporating soursop have been reported to exhibit more stable emulsions and better rheological properties (Virgen‐Ceceña et al. 2019).

#### 3.3.5 Moisture Content

The results of the effect of soursop puree and gum arabic incorporation on the moisture content of the ice cream are presented in Table 4. Moisture content in the ice cream increased from 53.30% to 64.96% as the levels of soursop puree incorporated increased. There was no significant difference (*p* > 0.05) in moisture content due to the addition of soursop puree at 10% and 20%. However, these results differed significantly (*p* < 0.05) from samples containing 30% soursop and those without soursop. The addition of gum arabic did not have a significant effect on the moisture content (*p* > 0.05). The lowest moisture content (56.24% ± 5.18%) was observed at 1% gum arabic, while the highest (59.04% ± 7.04%) was observed at 0.5% gum arabic. The effect of the interaction between soursop and gum arabic increased the moisture content range to between 50.57% and 69.71% (Table 5). In a study conducted by Perera and Perera (2021) to determine the physicochemical properties of spicy coconut milk–based ice cream, the obtained moisture content was 61.86% ± 0.33%. This value falls within the range of values found in the current study. In a different study by Winarsih et al. (2020), which utilized kidney nut puree in coconut milk–based ice cream, the moisture content ranged between 65.79% and 72.61%, slightly higher than the values obtained in the present study. Moisture in ice cream arises from the ingredients used, such as milk and cream. The incorporation of soursop also contributed to the increased moisture content. In ice cream manufacturing, moisture serves to dissolve solid ingredients such as sugar, milk powder, and stabilizers. However, higher moisture content in ice cream can lead to increased crystallization, which is undesirable for consumers (Buniowska‐Olejnik et al. 2023).

#### 3.3.6 Vitamin C Content

The vitamin C content was highest (22.66 mg/100 g) in the ice cream with 30% soursop incorporation and lowest (5.36 mg/100 g) in treatments without soursop (Table 4). There was a significant difference (*p* < 0.05) in vitamin C content among samples containing soursop puree and those without. However, there was no significant effect (*p* > 0.05) on the vitamin C content due to the addition of different levels of gum arabic. The vitamin C content increased up to 27.71 mg/100 g for treatments with 30% soursop and 0.5% gum arabic (Table 5). Soursop fruit is a rich source of vitamin C nutritionally (Bhat and Paliyath 2016). Akomolafe and Ajayi (2015) reported that soursop fruit pulp contains approximately 10.45 ± 0.11 mg/100 g of vitamin C, although the peel contains a considerably higher amount of about 19.29 ± 0.10 mg/100 g. As the demand for more nutritious foods rises, the incorporation of fruit pulps in frozen desserts is gaining popularity. Vitamin C in the diet acts as an antioxidant with oxygen‐scavenging activity, a property that ameliorates oxidative stress thus reducing the chances of cancerous cell growth. In a study by Ogo et al. (2021), ice cream was formulated from kunu zaki (millet milk) with banana, mango, and avocado pulps, resulting in a significant increase in vitamin C levels, with concentrations ranging from 18.79 to 38.42 mg/100 g and the highest level observed in the mango pulp ice cream. These results are comparable to what was obtained in the current study. Fruit pulps have been found to contain significantly high quantities of vitamin C, making them popular choices for incorporation into frozen desserts.

#### 3.3.7 Total Phenolic Content

The TPC ranged from 408.11 to 4332.53 mg GAE/100 g (Table 4). Each unit increase in soursop puree incorporation resulted in a statistically significant (*p* < 0.05) increase in the total phenolic content of the ice cream. Similarly, the addition of 1.5% gum arabic also resulted in the highest total phenolic content (1999.71 ± 1504.18 mg GAE/100 g). However, this was not significantly different (*p* > 0.05) from treatments with lower levels of gum arabic. The interaction effect between soursop puree and gum arabic also significantly (*p* < 0.05) increased the total phenolic content, ranging from 361.36 to 4378.40 mg GAE/100 g (Table 5). The TPC in the current study fell within the range reported in other studies that utilized fruit puree or plant extracts in the manufacture of ice creams. Gremski et al. (2019) reported a TPC ranging between 312 and 1701 mg GAE/L in a functional ice cream formulated with the addition of herbal tea extracts. Similarly, Gopika et al. (2022) reported a range of 300.23 to 800.48 mg GAE/100 g for the TPC in an ice cream containing powder extracted from marigold flower petals. However, the findings in this study are lower than those reported by Sayar et al. (2022) for camel milk ice cream with blueberry fruits, where the total phenolic content ranged from 6131 to 10,115 mg GAE/100 g at 5%, 10%, and 15% blueberry incorporation levels. Despite the current study using 30% soursop fruit pulp, the difference can be attributed to the higher total phenolic content in blueberries, reported at 67,690 mg GAE/100 g by Sayar et al. (2022). Polyphenols are bioactive compounds naturally found in plants, and they are abundant in fruits such as berries, grapes, and pears, as well as in products incorporated with these fruits (Rahman et al. 2021). Soursop fruit pulp is also a rich source of these phenolic compounds, quantified at 550.7 ± 0.33 mg GAE/g by Ogunkunle et al. (2020). This could explain the high TPC in the ice cream treatments containing high levels of soursop puree addition. The abundance of polyphenolic compounds in food is associated with high antioxidant activity, which, in turn, functions to protect the body from oxidative stress linked to conditions such as cancer, diabetes, cardiovascular diseases, and other related illnesses (Nardini 2022).

### 3.4 Effect of Soursop Puree and Gum Arabic Incorporation on the Mineral Content of Nondairy Coconut Milk–Based Ice Cream

The mineral composition of ice cream is influenced by the addition of functional ingredients, which can enhance its nutritional profile. In this study, the mineral content was evaluated to determine the effects of soursop puree and gum arabic, both individually and in combination. The impact of varying levels of soursop puree and gum arabic on the mineral profile of the ice cream is shown in Table 6, while Table 7 presents the interaction effects between these two ingredients. Overall, the addition of these components contributed to a wide range of mineral levels, with notable variations in potassium, zinc, calcium, magnesium, iron, and sodium. Potassium content ranged from 863.88 to 1199.15 mg/kg when soursop puree was added and 818.68 to 1203 mg/kg when gum arabic was added. On the other hand, zinc was established as the least abundant mineral in the ice cream, ranging from 4.58 to 6.33 mg/kg for different levels of soursop puree added and 4.80 to 5.95 mg/kg when gum arabic was incorporated. Magnesium and calcium content were comparably high because of different levels of the single factors. On the addition of soursop puree, calcium content ranged from 4.60 to 53.71 mg/kg, and magnesium content ranged from 45.51 to 84.84 mg/kg. Iron content ranged from 6.13 to 7.23 mg/kg, while sodium content ranged from 33.90 to 47.77 mg/kg. On the addition of gum arabic, calcium content ranged from 8.05 to 32.56 mg/kg, and magnesium content ranged from 47.41 to 97.90 mg/kg. Iron content ranged from 4.79 to 8.09 mg/kg, while sodium content ranged from 36.69 to 49.60 mg/kg (Table 6). The mineral content of the ice cream treatments due to the interaction between different levels of soursop puree and gum arabic is presented in Table 7. There was a significant effect (*p* < 0.05) due to the interaction between soursop puree and gum arabic that led to higher mineral content for zinc and sodium and the lowest for calcium and magnesium. For most minerals, higher values were obtained due to the interaction between 30% soursop puree and different levels of gum arabic. In the absence of soursop, most minerals displayed lower contents. On average, the control treatment displayed low mineral content for most minerals. Soursop fruit pulp is a rich source of minerals such as calcium (24.3 to 77 mg/kg), iron (1.40 to 10.15 mg/kg), zinc (0.36 to 6.37 mg/kg), sodium (49.2 mg/kg), magnesium (47.2 mg/kg), and potassium (544 to 1761 mg/kg), and therefore its incorporation into ice cream resulted in a mineral‐dense product (Inkalaba et al. 2023; Nascimento et al. 2019). In a study investigating the effect of enriching ice cream with different levels of peach peel and pulp powder, Yangılar (2016) reported a significant increase in calcium, iron, zinc, magnesium, and potassium concentration, while sodium levels decreased, although the decrease was not significant. The calcium content in the ice cream containing peach pulp ranged between 2101 and 2104 mg/kg, which was significantly higher than the results in this study. This difference was attributed to the high calcium content reported at 1121 mg/kg in bovine milk compared to 471 mg/kg in coconut milk, which was the base ingredient in the current study. This observation was similar for most minerals, with bovine milk expressing higher concentrations than plant‐based milks such as coconut (Walther et al. 2022). Similarly, it was observed that in most studies where fruit pulp was incorporated into dairy ice creams, most minerals displayed higher concentrations compared to when incorporated into nondairy ice creams. In a different study by Yangılar (2015), there was an observable increase in the mineral contents for every 1% increase in date fiber incorporated in the ice cream. According to Rejman et al. (2021), most fruits have been found to contain considerably high amounts of minerals that play vital roles in the body.

**TABLE 6:** Mineral content of ice cream due to soursop puree and gum arabic incorporation.

	Calcium	Iron	Zinc	Sodium	Magnesium	Potassium
*Soursop (%)*						
0	5.09 ± 0.62ᵇ	7.23 ± 0.63ᵃ	5.72 ± 0.78ᵇ	47.77 ± 5.61ᵃ	69.63 ± 4.90ᵇ	1010.07 ± 93.71ᵇ
10	6.03 ± 0.82ᵇ	6.89 ± 0.57ᵃ	4.85 ± 0.56ᶜ	45.61 ± 8.15ᵃ	45.51 ± 7.24ᶜ	1199.15 ± 115.75ᵃ
20	4.60 ± 0.64ᵇ	6.86 ± 0.65ᵃ	4.58 ± 0.61ᶜ	33.90 ± 1.38ᶜ	64.15 ± 4.98ᵇ	863.88 ± 109.81ᶜ
30	53.71 ± 13.17ᵃ	6.13 ± 0.92ᵇ	6.33 ± 0.79ᵃ	40.15 ± 4.57ᵇ	84.84 ± 17.42ᵃ	1077.89 ± 116.50ᵇ
*Gum levels (%)*						
0	32.56 ± 17.08ᵃ	7.42 ± 0.57ᵇ	5.82 ± 0.96ᵃ	36.69 ± 4.70ᶜ	61.21 ± 8.12ᵇ	1203.00 ± 104.66ᵃ
0.5	15.44 ± 6.48ᵇ	4.79 ± 0.24ᵈ	4.91 ± 0.62ᵇ	49.60 ± 5.88ᵃ	97.90 ± 13.12ᵃ	1020.32 ± 87.5ᶜ
1	8.05 ± 2.10ᶜ	8.09 ± 0.48ᵃ	4.80 ± 0.47ᵇ	39.04 ± 6.54ᶜ	57.61 ± 7.74ᵇ	818.68 ± 111.49ᵈ
1.5	13.37 ± 6.35ᵇ	6.81 ± 0.78ᶜ	5.95 ± 0.71ᵃ	42.10 ± 4.91ᵇ	47.41 ± 3.43ᶜ	1108.99 ± 116.72ᵇ

*Note:* Values are mean scores mg/kg ± standard error (*n* = 3). Means followed by different superscript letters in the same column are significantly different (*p* < 0.05).

**TABLE 7:** Mineral content of ice cream samples due to the interaction between soursop puree and gum arabic.

Soursop %	Gum%	Calcium	Iron	Zinc	Sodium	Magnesium	Potassium
0	0	7.22 ± 0.04ᵈ	7.21 ± 0.05ᶜ	2.76 ± 0.24ᶠ	25.96 ± 0.95ʰ	72.09 ± 0.21ᵇᶜᵈ	1412.71 ± 2.21ᵃᵇᶜ
	0.5	2.71 ± 0.08ᵈ	4.60 ± 0.05ᶠᵍ	5.28 ± 0.20ᵈ	47.74 ± 0.39ᵈ	88.43 ± 1.55ᵇ	942.73 ± 0.80ᵉ
	1	4.75 ± 0.15ᵈ	9.01 ± 0.18ᵃ	6.44 ± 0.27ᶜ	67.65 ± 1.64ᵇ	65.20 ± 2.23ᶜᵈᵉ	737.93 ± 20.50ᶠᵍ
	1.5	5.67 ± 0.32ᵈ	8.11 ± 0.42ᵇ	8.39 ± 0.25ᵇ	49.72 ± 1.34ᵈ	52.80 ± 1.34ᵉᶠ	946.91 ± 27.60ᵉ
10	0	7.36 ± 0.03ᵈ	5.77 ± 0.10ᵉ	6.84 ± 0.12ᶜ	57.49 ± 0.70ᶜ	34.87 ± 0.26ᶠᵍʰ	1536.03 ± 18.36ᵃ
	0.5	7.36 ± 0.30ᵈ	5.45 ± 0.08ᵉᶠ	2.76 ± 0.15ᶠ	74.97 ± 1.86ᵃ	75.26 ± 1.81ᵇ	1243.49 ± 25.13ᶜᵈ
	1	7.11 ± 0.16ᵈ	7.08 ± 0.32ᶜ	5.25 ± 0.00ᵈ	23.92 ± 0.08ʰ	24.06 ± 0.15ʰ	1313.54 ± 4.95ᵇᶜᵈ
	1.5	2.28 ± 0.08ᵈ	9.25 ± 0.06ᵃ	4.55 ± 0.06ᵈᵉ	26.08 ± 0.06ʰ	47.84 ± 0.16ᵉᶠᵍ	703.54 ± 1.20ᵍʰ
20	0	5.13 ± 0.19ᵈ	6.85 ± 0.02ᶜ	4.27 ± 0.13ᵉ	28.96 ± 0.44ᵍʰ	47.82 ± 1.11ᵉᶠᵍ	906.14 ± 21.39ᵉᶠ
	0.5	7.11 ± 0.19ᵈ	5.13 ± 0.09ᵉᶠ	7.21 ± 0.07ᶜ	35.24 ± 0.85ᵉᶠ	73.94 ± 1.43ᵇᶜᵈ	706.24 ± 15.97ᵍʰ
	1	3.01 ± 0.04ᵈ	9.59 ± 0.23ᵃ	3.01 ± 0.04ᶠ	37.57 ± 0.47ᵉᶠ	79.14 ± 1.98ᵇᶜ	540.37 ± 15.97ʰ
	1.5	3.15 ± 0.31ᵈ	5.88 ± 0.42ᵉ	3.83 ± 0.54ᵉ	33.82 ± 3.50ᶠᵍ	55.70 ± 5.43ᵉ	1302.79 ± 112.73ᵇᶜᵈ
30	0	110.56 ± 7.71ᵃ	9.84 ± 0.28ᵃ	9.40 ± 0.08ᵃ	34.35 ± 1.63ᶠᵍ	90.05 ± 4.74ᵇ	957.15 ± 49.32ᵉ
	0.5	44.57 ± 5.59ᵇ	3.99 ± 0.58ᵍ	4.38 ± 0.51ᵉ	40.46 ± 5.34ᵉ	153.97 ± 22.22ᵃ	1188.83 ± 175.01ᵈ
	1	17.32 ± 0.14ᶜ	6.68 ± 0.13ᶜᵈ	4.52 ± 0.13ᵈᵉ	27.01 ± 0.11ʰ	62.04 ± 1.33ᶜᵈᵉ	682.90 ± 5.40ᵍʰ
	1.5	42.39 ± 0.48ᵇ	4.00 ± 0.33ᵍ	7.04 ± 0.35ᶜ	58.77 ± 0.68ᶜ	33.31 ± 1.19ᶠᵍʰ	1482.70 ± 11.80ᵃᵇ

*Note:* Values are mean scores (mg/kg) ± standard error (*n* = 3). Means followed by different superscript letters in the same column are significantly different (*p* < 0.05).

## 4 Conclusion

In conclusion, the incorporation of fruit pulps and plant gums into ice cream has proven to be a promising innovation, particularly with the use of gum arabic, which effectively stabilizes and emulsifies coconut milk–based ice cream. This study demonstrated that higher concentrations of soursop puree, combined with gum arabic, significantly enhanced the ice cream's overrun and reduced meltdown time, resulting in a creamier texture. Gum arabic plays a crucial role in increasing total soluble solids (TSS), which improves the body and whipping ability of the ice cream mix. Moreover, the addition of soursop puree affects the pH levels, contributing to a more appealing structure through desirable acidity. Although increasing the amount of soursop puree has a significant impact, it is essential to limit the use of gum arabic to 1.5% to maintain optimal mix viscosity and the overall quality of the ice cream. Maintaining moisture balance during ice cream production is also critical to prevent unwanted crystallization, which can affect the final product's texture. The nutritional profile of frozen desserts can be significantly enhanced by carefully selected ingredients; in this case, soursop puree not only elevates vitamin C levels but also alters the mineral content of the ice cream. The findings of this study indicate that the combination of 30% soursop puree with varying amounts of gum arabic resulted in the highest mineral concentrations, particularly zinc and sodium, while reducing levels of calcium and magnesium. Additionally, the high polyphenolic content in soursop fruit pulp suggests potential health benefits due to increased antioxidant activity in these ice cream formulations. Overall, the combination of soursop puree and gum arabic in coconut milk–based ice cream not only improves its nutritional value but also enhances its quality attributes, offering exciting possibilities for the development of healthier frozen desserts.

## Author Contributions

**Sarah Nura Mulwa:** conceptualization (lead), data collection (lead), data analysis (lead), writing – original draft (lead). **Symon Maina Mahungu:** conceptualization (supporting), supervision (supporting), writing – review and editing (supporting). **Benard Kimatu Muinde:** conceptualization (supporting), supervision (supporting), writing – review and editing (supporting).

## Conflicts of Interest

The authors declare no conflicts of interest.

## Data Availability

The data that support the findings of this study are available from the corresponding author upon reasonable request.
